# Quality of Life in Patients with NSCLC Receiving Maintenance Therapy

**DOI:** 10.3390/cancers7020817

**Published:** 2015-05-29

**Authors:** Achim Rittmeyer

**Affiliations:** Lungenfachklinik Immenhausen, Thoracic Oncology, Immenhausen 34376, Germany; E-Mail: arittmeyer@lungenfachklinik-immenhausen.de; Tel.: +49-56-7350-1421; Fax: +49-56-7350-1319

**Keywords:** HRQoL, NSCLC, maintenance therapy

## Abstract

*Introduction*: In the past few years many trials have evaluated the use of maintenance therapy in the treatment of NSCLC stage IV. Both switch as well as continuation maintenance show an improved PFS and overall survival. HRQoL data was only partially published. The aim of this article is to review the published effects of maintenance therapy on HRQoL. *Methods*: Two PubMed searches were performed using the terms: “maintenance therapy and NSCLC” and “maintenance therapy and NSCLC and HRQoL”. The published data was compared, analysed and evaluated. *Results*: 272 articles were found dealing with maintenance therapy, and of these 85 articles were found regarding maintenance therapy and HRQoL in NSCLC. Maintenance therapy showed no negative impact on HRQoL but failed to show a real benefit. Some symptoms showed positive trends during maintenance therapy. HRQoL can be used to select patients for maintenance therapy. *Conclusions*: Maintenance therapy is very safe, improves PFS and OS without impairing HRQoL. Although a positive impact on general QoL could not be demonstrated this is possibly due to the mode of evaluating HRQoL. Patient reported outcomes should be simplified and examined for a longer period of time.

## 1. Introduction

Lung cancer remains the leading cause of cancer death worldwide, with only small improvements by first- and second-line chemotherapy. Median overall survival (OS) of patients with metastatic NSCLC in trials still ranges below the one year threshold, exceeding this threshold only in selected populations, e.g., with activating mutations or those deemed fit enough to be treated with cisplatin and bevacizumab [1].

Accordingly, strategies to improve survival are still warranted. One of the most thoroughly evaluated strategies in the last years was maintenance therapy. All maintenance trials share the effort to prolong first-line-therapy usually until tumour progression or inacceptable toxicities occur [2]. Two different approaches have been used. First, to change the drug after four cycles of first-line-therapy e.g., erlotinib after four cycles of platinum-based doublet (“switch maintenance”) [3,4]. Second, to continue at least one compound of the first-line-combination beyond the fourth cycle, e.g., continue pemetrexed after four cycles of pemetrexed/cisplatin (“continuation maintenance”) [5,6].

Both strategies were able to prolong progression free survival PFS and OS [2]. Although the effect on overall survival was quite robust and exceeding the amount often seen in other first-line-trials in NSCLC, there remained a controversy as to whether this survival benefit was clinically meaningful [7]. To improve health related quality of life (HRQoL) is one factor to identify therapeutic changes as clinically meaningful [8]. Even patient selection for maintenance therapy could possibly be guided by assessment of QoL [9]. The aim of this article is to review the published effects of maintenance therapy on HRQoL.

## 2. Methods

Two PubMed searches were performed. One using the terms: “maintenance therapy and NSCLC” another “maintenance therapy and NSCLC and HRQoL”. For further evaluation only fully published, original, phase III data using (at least temporarily) approved drugs was considered.

Only trials starting with platinum based therapy were defined as maintenance trials as all TKIs are given until disease progression or unacceptable toxicity occur, but are usually not called maintenance therapy if given as first-line-therapy.

As patient reported outcome does often not agree with investigator reported measurements with minor exceptions, only patient reported HRQoL was considered, not physician reported adverse events or performance status [10].

## 3. Results

272 articles were found dealing with maintenance therapy and 85 articles were found regarding maintenance therapy and HRQoL in NSCLC. The ECOG 4599 and the AVAIL trials both using bevacizumab as a maintenance therapy were excluded as there was no comparison for the bevacizumab maintenance, but only a comparison between using bevacizumab from the start *vs.* not using bevacizumab at all [11,12].

Ten fully published phase III trials using different types of maintenance therapy in metastatic NSCLC were found and are listed in Table 1. Eight of these have published health related quality of life reported by patients (PRO) until now. Two trials published survival and HRQoL data together and six trials published their HRQoL data separately, revealing more data.

**Table 1:** Fully published phase III trials using maintenance therapy in patients with NSCLC.

First Author	Induction *n*	Maintenance *n*	% Maintenance	Regimen Used	Maintenance	Survival Difference	HRQoL (PRO)
Brodowicz [13]	352	206	58.5%	Gemcitabine/cisplatin followed by gemcitabine or BSC	Continuation gemcitabine	TTP *p* < 0.001	LCSS trend favours better control of haemoptysis, cough and pain in maintenance arm
						OS 13.0 *vs.* 11.0 mon	
						*p* = 0.195	
Ciuleanu [4,9,14]	Na	663	na	Four cycles platinum based doublet followed by pemetrexed	Switch Pemetrexed	PFS 4.4 *vs.* 2.6 mon	LCSS published separately significant longer time to worsening pain and haemoptysis
						HR 0.50 *p* < 0.0001	
						OS 13.4 *vs.* 10.6	
						HR 0.79 *p* = 0.012	
Cappuzzo [3,15]	1949	889	45.6%	Four cycles platinum based doublet followed by erlotinib or placebo	Switch Erlotinib	PFS 12.3 *vs.* 11.1 weeks	FACT-L time to deterioration no difference in HRQoL
						HR 0.71 *p* < 0.0001	
						OS 12.0 *vs.* 11.0 mon	Time to pain improved by maintenance erlotinib but not time to cough or dyspnoea
						HR 0.81 *p* = 0.009	
Paz-Ares [6,16,17,18]	939	539	57.4%	Pemetrexed/cisplatin followed by pemetrexed or placebo	Continuation Pemetrexed	PFS 4.4 *vs.* 2.8 mon	EQ5-D published separately
						HR 0.60 *p* < 0.01	
						OS 13.9 *vs.* 11.0	No significant differences, but Qol improvement by induction-cycles
						HR 0.78 *p* = 0.02	
Westeel [19]	573	227	39.6%	Four cycles of MIC followed by vinorelbine maintenance or observation	Switch Vinorelbine	PFS 5.0 *vs.* 3.0	Not published
						HR 0.77 *p* = 0.11	
						OS 12.3 *vs.* 12.3	
						HR 1.08 *p* = 0.65	
Sculier [20]	485	281	59.6%	Three cycles of GIP gemcitabine/ifosfamid/cisplatin followed by paclitaxel or GIP	Switch	PFS 4.0 *vs.* 4.4	Not published
					Paclitaxel	*p* = 0.56	
					Continuation	OS 9.7 (Pacli) *vs.* 11.9 (GIP)	
					Gemcitabine	HR 0.81 *p* = 0.10	
Barlesi [5,21,22]	376	253	67.3%	Pemetrexed/cisplatin/bevacizumab followed by pemetrexed/bevacizumab or bevacizumab alone	Continuation	PFS 7.4 *vs*. 3.7 mon	EORTC QLQ-C30 and QLQ-LC13 Published separately no significant differences but trend to better pain and dyspnoea control in combination arm
						HR 0.48, *p* < 0.001	
					Pemetrexed and bevacizumab	OS 17.1 *vs.* 13.2	
						HR 0.87 *p* = 0.29	
Perol [23]	834	464	55.6%	Gemcitabine/cisplatin followed by gemcitabine or erlotinib maintenance compared to BSC	Continuation	Gem PFS 3.8 *vs.* 1.9 mon	Not published
					Gemcitabine	HR 0.56 *p* < 0.01	
					Switch	OS 12.1 *vs.* 10.8	
					Erlotinib	HR 0.89 *p* = 0.39	
Zhang [24,25]	Na	296	Na	Four cycles platinum based doublet followed by gefitinib or placebo	Switch	PFS 4.8 *vs.* 2.6	FACT-L compliance rate 47% (gefitinib) and 33% (placebo) only 10% known EGFR-Mutation status
						HR 0.42 *p* < 0.0001	
					Gefitinib	OS 18.7 *vs.* 16.9	
						HR 0.84 *p* = 0.26	
Patel [26,27]	Na	939	Na	Pemetrexed/carboplatin/bevacizumab followed by pemetrexed/bevacizumab *vs.* paclitaxel/carboplatin/ bevacizumab followed by bevacizumab alone	Continuation	PFS 6.0 *vs.* 5–6 mon	FACT-L without differences between both arms
					Bevacizumab alone	HR 0.83, *p* = 0.012	
					Continuation	OS 12.6 *vs.* 13.4 mon	FACT-Ntx favouring pemetrexed containing arm regarding neurotoxicity
					Pemetrexed and bevacizumab	HR 1.0 *p* = 0.95	

The QoL-assessment of the INFORM-trial was not further addressed by this article as the compliance rate for the FACT-L questionnaires used in this trial was only between 33% and 47%. Given the fact that at the time the trial was recruiting patients (September 2008–August 2009) the IPASS data was already available a known EGFR-Mutation-Status of only 10% in a purely East Asian population is likely to mix up the effects of maintenance therapy with those of effective TKI therapy [24].

Also the pointbreak trial was excluded from further evaluation as the primary focus of the trial was not maintenance therapy, but rather to compare two different regimens from the start. There is little to no HRQoL data available to compare only the two different maintenance regimens used in the pointbreak trial [26,27]. Socinski compared continuous to four cycles of carboplatin paclitaxel but with an upfront randomization and was also not regarded as a maintenance trial [28].

Brodowicz *et al*. were the first to use a maintenance regimen discontinuing the platinum compound after four cycles and using gemcitabine as a partner of cisplatin as continuation maintenance [13]. He used the LCSS to evaluate HRQoL, but published only five lines of narrative without any figures or numbers indicating that no significant difference was detected other than a trend towards better control of cough, haemoptysis and pain, as well as for the LCSS-total score [13]. However, Brodowicz gave a more detailed analysis regarding the Karnofsky performance status (KPS). In patients with a KPS > 80% he found a OS of 22.9 months for patients with gemcitabine maintenance, compared to 8.3 months with BSC (HR 2.1 for BSC, no *p*-value given). If the KPS was <80% the OS was 7.0 *vs.* 7.7 months for gemcitabine maintenance and BSC, respectively (HR 0.8 for BSC) [13].

The LCSS was also employed by the investigators of the JMEN study using pemetrexed switch maintenance after four cycles of platinum-based doublet without pemetrexed. Worsening of symptoms was defined as a 15 mm increase from baseline. A statistically significant longer time to worsening of symptoms was detected for pain and haemoptysis, but not for the other seven scores of the LCSS. Generally a high rate of censored patients had to be admitted (54%–90%) which lowered the statistical power to detect differences. Only 48% in the pemetrexed arm and 54% in the placebo arm completed the assessment after treatment discontinuation [14].

The investigators of the JMEN trial published data that also supports the possibility of selecting patients who would benefit most from maintenance therapy [9]. They used only the six LCSS symptom scores, leaving out the three global scores, and calculated the mean value of the six scores, each ranging from 0 to 100. Thus they distinguished between a group of patients with low symptom burden (mean value < 25) and high symptom burden (mean value > 25) and could show that patients with a low symptom burden had an OS of 17.5 months when treated with pemetrexed maintenance compared to 11.0 months in the placebo group (HR 0.63, *p* 0.0012). In the high symptom group the OS was 11.8 months for pemetrexed maintenance and 10.6 months for placebo, respectively. (HR 1.02, *p* = 0.92) [9]. By using the ECOG performance status (PS) they could also find a similar difference with an OS in patients who had a PS 0 of 17.7 months for pemetrexed maintenance compared to 10.3 months for placebo (HR 0.54, *p* = 0.0019). In patients who had a PS of 1 the difference was only 14.1 to 10.6 months for pemetrexed maintenance and placebo, respectively (HR 0.78, *p* = 0.105).

In the Saturn trial the switch maintenance using erlotinib was compared to placebo after four cycles of several possible platinum doublets. The investigators also used the FACT-L score and could show no significant differences (HR 0.96) for the time to deterioration. But a *post hoc* analysis showed a significantly prolonged time to pain occurrence (HR 0.61, *p* = 0.008) and a non-significant trend toward a longer time to cough and dyspnoea (HR 0.77 [0.26] and 0.75 [0.21], respectively). The completion rate was ≥90% in both treatment-groups and at all study visits [15].

**Figure 1:**
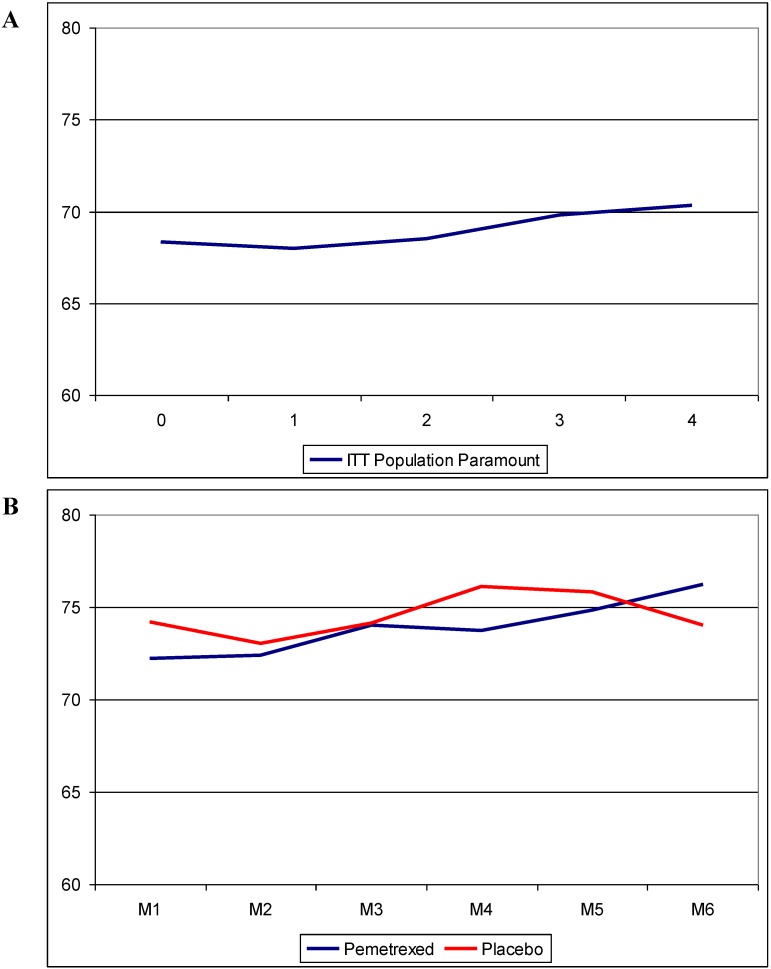
(**A**): HRQoL in the ITT population of the Paramount trial during four cycles of induction pemetrexed/cisplatin; (**B**): HRQoL in the maintenance population of the Paramount trial during six cycles of maintenance therapy. Modified from [29].

The Paramount investigators comparing pemetrexed continuation maintenance with placebo published their QoL-data using the EQ-5D questionnaire. They could show a statistically significant but not clinically meaningful improvement in QoL at induction cycle 3 and 4 of the ITT population (Figure 1A). No differences between the two treatment-arms could be detected in the maintenance phase of the trial. (Figure 1B) In both arms a clinically meaningful, but statistically non-significant, deterioration could be revealed at the 30 day post discontinuation visit but again with no significant difference between both arms. The compliance rate was 84.3% for the pemetrexed arm (1834 assessments completed of 2176 visits) and 80.9% for the placebo arm (807 assessments completed of 998 visits) [29].

The Avaperl-HRQoL data was also published separately. The investigators used the EORTC-QLQ-C30 and the QLQ-LC-13. Distribution rate of questionnaires was 96.8% and 98.4% at baseline decreasing to 71.4% (Bev alone) and 83% (pemetrexed plus bevacizumab) respectively at maintenance cycle 11. Completion rates were 80% for bevacizumab alone and 86% for pemetrexed plus bevacizumab, respectively [22].

**Figure 2:**
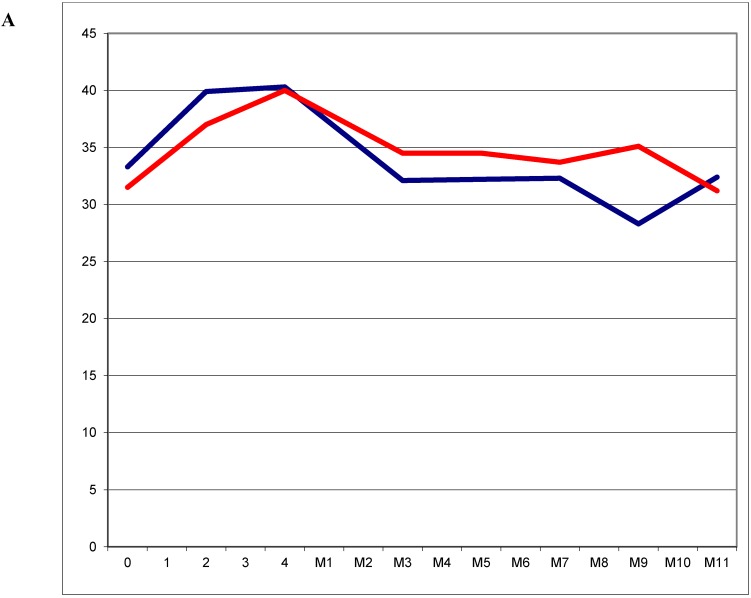

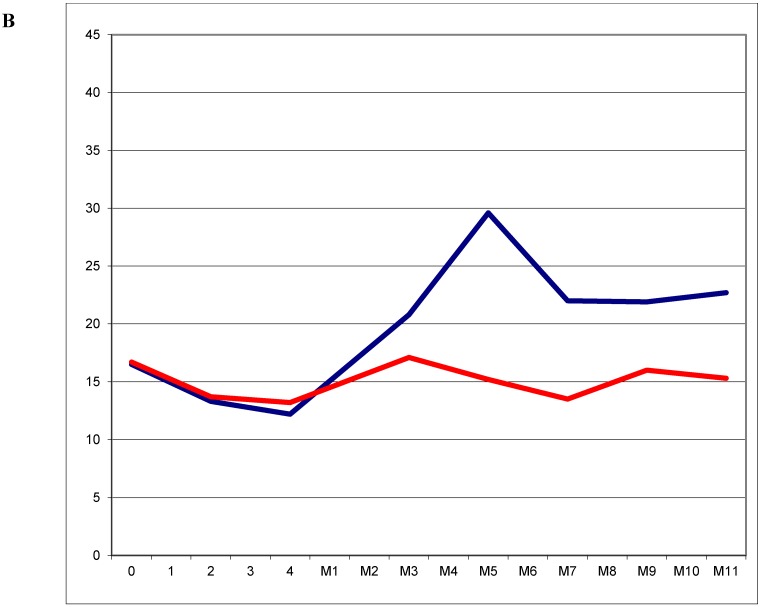
(**A**): Symptom score for fatigue in the maintenance population of the Avaperl trial during four cycles off induction pemetrexed/cisplatin/bevacizumab (0–4) and eleven cycles of maintenance (M1–11)with either bevacizumab alone (**blue line**) or pemetrexed plus bevacizumab (**red line**); (**B**): Symptom score for pain in the maintenance population of the Avaperl trial during four cycles off induction pemetrexed/cisplatin/bevacizumab and eleven cycles of maintenance with bevacizumab alone (**blue line**) or pemetrexed plus bevacizumab (**red line**) Modified from [22].

In contrast to the Paramount trial the investigators focused on the maintenance population beginning from the baseline assessment before induction-therapy was started. A slight decrease of cough and a slight increase in fatigue (Figure 2A) and appetite loss could be detected during induction therapy. In the maintenance phase of the trial coughing remained relatively low, whereas fatigue improved again after completion of induction therapy. Appetite-loss remained quite remarkable in the combination arm leading to a statistically significant and clinically meaningful difference at maintenance cycle 7 to 11 favouring bevacizumab alone. On the other hand pain control was favourable in the combination arm with statistically and clinically meaningful differences at maintenance cycle 9 and 11 (Figure 2B). Comparing the global health and functional scores no statistically or clinically meaningful differences could be detected throughout the whole trial.

## 4. Discussion

OS in metastatic NSCLC is prolonged by switch maintenance as well as by continuation maintenance. The amount of survival difference especially in continuation maintenance therapy is quite amazingly in the range of three months, considering the data of the Paramount trial and possibly even higher in the Avaperl trial [5,6,17,21]. As patients with metastatic lung cancer are doomed to die inevitably HRQoL is a key target for the thoracic oncologist [7]. After reviewing the present data dealing with QoL in maintenance therapy of NSCLC five conclusions can be drawn.

### 4.1. Keep it Short and Simple! Different Tools to Assess QoL

A key issue to understand the published QoL data in maintenance therapy is to examine the tools used to investigate HRQoL. The FACT-L questionnaire consists of 37 questions allowing five degrees each [30]. This leads to 185 possible answers at each point of time. The EORTC QLQ-30 enlarged by the QLQ-LC13 [31,32] leads to 172 possible answers at any point of evaluation.

It is dubious if patients suffering from incurable lung cancer will scrutinize each question and answer these questions in the way the questionnaire was originally designed. This leads to uncertainties as to how reliably the questions are answered by the patients. Also the investigators might find it difficult to get a clear picture as the QoL-data is summed up in a whole bunch of scores with largely overlapping values.

Finally the risk to see statistically significant results just by chance is high if you perform very many tests, e.g., for the HRQoL of the Avaperl trial eight functional scores were examined and an additional eight symptom scores every cycle from baseline before the first cycle of induction therapy until the 11th cycle of maintenance therapy, so basically 240 scores were received post baseline and just by chance one should find 12 statistically significant values using the usually employed p value of 0.05.

This has led to the recommendation to use only a certain amount of differences as a clinically meaningful result. These clinically meaningful results have been evaluated as the smallest difference that patients perceive as beneficial e.g., ≥10 points in the various scores derived from the EORTC-questionnaires [31,33,34].

The EQ-5D and the LCSS are easier questionnaires for the patients as well as for the interpreting physicians. The EQ-5D asks only five questions with three degrees each but the questions asked in the EQ-5D are not specifically targeted to the lung cancer population. Another part of the EQ-5D is the VAS (visual analog scale) part of the questionnaire. The patients have to rate their actual QoL on a scale from 0 (worst imaginable health) to 100 (best imaginable health).

The first part of the LCSS consists of nine VAS (six symptoms and three general scores). This makes it quite easy for the patients to complete the questionnaire quickly and without having to decide whether they feel “only a bit”, “somewhat” or “quite a bit” (FACT-L) distressed in any symptom as they can choose graphically on a 10 cm range, but it makes it difficult to impossible for the patients to remember what they answered the last time making it difficult to weigh changes in symptoms over time. The second part of the LCSS has to be answered by the investigator and consists of six items with five degrees each [35,36].

Each of the four trials with separately published extensive HRQoL data used a different questionnaire (Saturn LCSS, JMEN FACT-L, Paramount EQ-5D, Avaperl EORTC QLQ 30 and LC13). Obviously the optimal tool to measure HRQoL has not yet been found, and there is no general agreement as to which tool should be recommended for future trials.

### 4.2. Some Symptoms Can Be Improved and Controlled for a Longer Period of Time by Maintenance Therapy

Regarding the general HRQoL none of the trials could show a significant or clinically meaningful improvement. Nevertheless there are still some indications of a small improvement through maintenance therapy. At least focussing on certain symptoms e.g., pain is improved in erlotinib switch-maintenance (Saturn) [15] gemcitabine continuation maintenance [13] and pemetrexed plus bevacizumab maintenance (Avaperl) [22]. Dyspnoea is one of the most frightening symptoms for patients suffering from lung cancer and there was a trend towards a better dyspnoea control using gemcitabine continuously [13] or pemetrexed plus bevacizumab continuously [22], but not in erlotinib or pemetrexed switch-maintenance [14,15].

### 4.3. Not All Symptoms Are Equal

Fatigue in general seems to be a more distressing symptom than pain as can be seen in the Avaperl trial (comparing Figure 2A,B) [22] and as well in the JMEN trial looking at the baseline scores of 33.3–33.9 for fatigue and 14.8–15.5 for pain. Even dyspnoea seems to be less distressing than fatigue [14]. This is important to notice as dyspnoea and pain can be treated much more easily than fatigue.

### 4.4. PS and Symptoms Can Be Used to Select Patients Who Benefit from Maintenance Therapy

Just by measuring the symptom burden of their patients the JMEN investigators could distinguish patients who could benefit most from maintenance therapy [9]. This has not been examined by any other trial. But this data is also supported by Brodowicz who could show a positive therapeutic effect of continuous gemcitabine only in patients with Karnovsky PS > 80 after induction therapy [10]. The JMEN investigators themselves also showed PS to be of predictive value after induction therapy so again more complicated does not mean better. The PS after induction therapy is very easy to assess and does help to select patients for maintenance therapy.

### 4.5. QoL Should Be Assessed Much Longer

At first sight it appears to be a smart idea to look at time to deterioration of QoL as you might expect a longer time to worsening of symptoms if your PFS increases. Accordingly a prolonged time to pain could be detected in the SATURN trial, but in general no significant differences could be detected [15].

The reason might simply be that the radiographic progress leading to discontinuation precedes any clinically meaningful worsening of symptoms by many weeks. E.g., in the JMEN trial a median number of five cycles of maintenance therapy was delivered in the pemetrexed arm, the fifth cycle was delivered only 12 weeks (around about three months) after baseline. The most probable reason for ending maintenance therapy was progressive disease *i.e.*, radiographic progression [4], but after a median time of 3.1–6.5 months after baseline the LCSS scores were censored due to no worsening at this point of time [14], so despite radiographic progression, no deterioration in clinical symptoms could be detected.

All the published HRQoL data has been collected only during treatment and once about 30 days after discontinuation. Given the fact that e.g., in the Paramount trial patients live three months longer one has to admit that there will be a difference in HRQoL between patients dying and those going on to live for another three month.

One strong suggestion of this review is to assess QoL as simply as possible for as long as possible. Preferably QoL assessment should go on as close to death as possible at least over more lines of therapy. If we evaluate QoL without any specific question, using extremely complicated tools and only during one line of treatment we are likely to miss clinically very meaningful information that could help us choose the right therapy for the right patient.

## 5. Conclusions

Maintenance therapy is very safe and improves PFS and OS significantly. In general no statistically or clinically significant impact, either positive or negative on HRQoL could be detected but some symptoms like pain, cough and dyspnoea tend to be better and controlled longer if maintenance therapy is used. In future studies HRQoL should be assessed for a longer period of time if possible until death. The tool used to assess QoL should be short and simple.
